# Patterns of Food Selectivity among Children with Autism Spectrum Disorder

**DOI:** 10.3390/jcm12175469

**Published:** 2023-08-23

**Authors:** Anna Byrska, Idalia Błażejczyk, Anna Faruga, Maria Potaczek, Krzysztof M. Wilczyński, Małgorzata Janas-Kozik

**Affiliations:** 1 Student’s Scientific Association, Department of Psychiatry and Psychotherapy of Developmental Age, Medical University of Silesia, 40-055 Katowice, Poland; 2 John Paul 2nd Child and Family Health Center in Sosnowiec Sp. z o.o., 41-218 Sosnowiec, Poland; 3 Department of Psychiatry and Psychotherapy of Developmental Age, Medical University of Silesia, 40-055 Katowice, Poland

**Keywords:** autism spectrum disorder, food selectivity, sensory sensitivity, children

## Abstract

Autism spectrum disorder (ASD) is a heterogeneous group of neurodevelopmental disorders characterized by food selectivity in a significant portion of the population. The nature of this selectivity remains unclear, with hypotheses suggesting associations with sensory disorders or stereotypical and repetitive patterns of activity and interests. This study aimed to determine the prevalence and nature of food selectivity traits in individuals with ASD compared with the neurotypical population. This study involved 219 participants, with 115 diagnosed with autism and 92 without. Twelve children undergoing diagnosis were excluded from the analyses. The findings revealed that food selectivity traits are more common in individuals with ASD, with differences in preferences mainly involving structure, color, taste, and serving method. Children with ASD had more food selectivity traits than those without, and the intake of certain food characteristics could be altered as they grow. Selectivity occurred for both sensory and stereotypical reasons, but stereotypical features significantly differentiated neurotypical individuals from those with ASD.

## 1. Introduction

Autism spectrum disorder (ASD) is a heterogeneous group of neurodevelopmental disorders manifested by the presence of qualitative and quantitative deficits in the creation and maintenance of interpersonal relationships, social communication, and repetitive and stereotyped behaviors and activities [1]. In the latest editions of the ICD-11 (2019) and DSM-5 (2013) classifications, nosological units formerly functioning separately within one diagnosis, namely, ASD, have been unified.

Data from the Centers for Disease Control and Prevention (CDC, 2023) reported that in 2020, 1 in 36 children aged 8 years was diagnosed with ASD [2]. This represents a sharp increase in the number of diagnoses since 1985, when the ratio was 1:2500 children. Later data from 1995 indicate the occurrence of ASD at a frequency of 1:500, while in 2005, it was 1:150 [2]. It is suggested that the increase in the frequency of ASD diagnoses may be due to unspecified environmental factors or a change in the diagnostic paradigm and a gradual widening of the existing criteria. The latter reason is probably also responsible for the difference in the incidence of ASD between the sexes, where the ratio of boys to girls was 43.0:11.4 [2]. The involvement of the NLGN4 gene is also postulated as the cause of “sex bias” observed in ASD [3]. A separate issue to remember is the hypothesis of the common occurrence of autistic traits in the general population, which postulates a departure from the dichotomous division into children without diagnosis and neuroatypical people.

The cause of autistic traits, despite many studies and hypotheses, remains unclear [1]. Autistic traits are characterized by sensory sensitivity; refusal to eat food for the first time; and food restrictions, for example, texture sensitivity, product brands, and problems with feeding [4]. Nevertheless, literature sources agree that they are primarily genetically determined, and their clinical presentation is subject to significant shaping elements of these environments [5]. There is a notably high concordance rate of ASD in monozygotic twins (92%), in contrast with a significantly lower rate in dizygotic twins (10%). While numerous genetic associations with ASD were uncovered, precise diagnostic markers remain elusive. Examples of genes associated with autism spectrum disorder include SHANK3, MECP2, CHD8, and FOXP2. Some evidence also highlights the role of epigenetic alterations, such as DNA methylation, in the development of ASD [6]. One of the hypotheses suggests the occurrence of intestinal microbiota disorders in children diagnosed with autism spectrum disorder, both in terms of quantitative and qualitative abnormalities (such as an increased colony size of, e.g., *Streptococcus* and *Clostridium*, and *Candida* fungi with a reduced amount, e.g., *Pseudomonas* or *Citrobacter*) [7]. However, the direction of this type of dependence is not clear. Some authors suggest that dysbiosis may be the basis of some of the disorders observed in children with ASD.

Meanwhile, from the available literature on healthy siblings, the microbiota profile was intermediate between the subjects and the population not affected by the disorder [8]. This, in turn, may suggest an inverse relationship in which ASD and the food selectivity observed in their course lead to microbiome abnormalities. The difference in the siblings’ microbiome would then result from the family’s diet adapted to the food selectivity of the child with autism, which has been enriched with deviations in the form of, for example, snacks.

Such reports and studies direct the interest of scientists toward the impact of a proper diet on the clinical picture of ASD. One of the areas in this type of analysis is the use of elimination diets. In 2009, Mulloy et al. [9] conducted a systematic review of the effect of gluten- and casein-eliminating diets on ASD symptoms. It showed that the majority of studies that presented a positive effect of nutritional interventions on ASD symptoms were of poor methodological quality. The rest of the studies analyzed showed negative or mixed effects. None of the papers gave conclusive results [8,9]. Despite the lack of convincing evidence, it is currently estimated that up to 26% of children with ASD are on a casein- and gluten-free diet, regardless of the existence of objective indications for its implementation [8], which can also significantly affect the composition of the intestinal microflora. Notwithstanding, in the case of patients with food intolerances, the use of elimination diets may bring some clinical effects. However, they are not related to the ASD itself, but rather to the coexisting alexithymia in this group of people, which according to the literature, may affect around 55% of the ASD population.

The concept of alexithymia refers to problems with recognizing and distinguishing between emotions and sensations coming from the body, difficulties in expressing emotions, and a deficit of imagination. People with alexithymia may have difficulties in identifying and understanding the source of unpleasant gastric symptoms [10], which are very common in people with ASD, although not secondary to that diagnosis, and depending on the source, occur in 9 to 90% of neuroatypical children. This relationship may be confirmed by a strong correlation between the severity of autism symptoms and the severity of gastrointestinal symptoms [11]. In addition, the discomfort resulting from the ailments may generate behavioral disorders and difficulties in functioning, which may be wrongly perceived as elements of the clinical picture of ASD.

A significant intersection is evident between the dysregulations of the autonomic nervous system in ASD [12]. This system is pivotal in controlling both digestive functions [13] and emotional responses [14]. It is known that dysautonomia can impact gut motility [15], potentially explaining some of the feeding difficulties and selectivity observed in individuals with autism. Emotional regulation, which is a process significantly influenced by the autonomic nervous system, may also be involved, as individuals with ASD often have difficulty processing emotional responses [16], which could further affect their eating behaviors.

One of the key symptoms of ASD is repetitive and stereotyped patterns of interests and activities (RRB), which include adherence to rituals, the need for consistency, and poor tolerance for new and unfamiliar activities and sensations. The DSM-5 also lists sensory integration disorders within the RRB domain. Originally described by Jean Ayres, they consist of the inability to organize the stimuli reaching the nervous system from the outside and integrate them with past experiences. As a consequence, hypersensitivity to noise, unpleasant tastes, smells, and tactile stimuli occurs [17].

Hypersensitivity to specific food characteristics, such as taste, color, temperature, or texture, leads to food selectivity [5,18,19]. Disorders of chewing food associated with unpleasant sensations during biting and with impaired muscle tone lead to impaired coordination of chewing and swallowing. As a consequence, there are serious dietary restrictions [20]. It was suggested that food selectivity is more common in children with autism spectrum disorders and may be associated with nutritional deficiencies [17,18]. Food selectivity may also occur in typically developing children, but in the case of patients with ASD, the repertoire of food intake is more limited [19,20]. Refusing foods with specific characteristics or reluctance to try new foods may occur with varying frequency in many patients and may interfere with the child’s daily functioning and integration in the peer group [21].

The results of studies by several authors indicate that children with ASD have significantly noticeable food selectivity compared to children without ASD, which may result in nutrient deficiency [22,23,24]. Considering how strongly dietary issues are related to the clinical picture and the functioning of people with ASD, it was of interest how the food preferences of children on the spectrum are shaped. Previous studies of food selectivity in the neuroatypical population suggest the need to investigate factors related to food selectivity, such as sensory integration disorders, behavioral disorders, or family food preferences [25,26].

## 2. Materials and Methods

The presented study was conducted using the authors’ questionnaire addressed to parents/guardians of children. Demographic questions regarded the age and sex of the child, the place of residence, and whether the examined person had a diagnosis of ASD. The main part of the questionnaire consisted of 37 items that divided foods into categories according to their crunchiness, appearance, taste, smell, temperature, color, moisture, consistency, degree of mixing of ingredients, consumption of vegetables, fruits, vitamins and supplements, and willingness to try new dishes. The aim of the study was to determine how willingly children eat food according to a specific characteristic of it on a 5-point scale, namely, does not eat at all, eats reluctantly, has no influence, eats willingly, eats very willingly, in order to check differences in the desire to eat food. The last part of the questionnaire was a non-obligatory open question about the child’s unusual behaviors related to nutrition. The questionnaire can be downloaded as Supplementary Table S1.

Between May 2021 to December 2021, both electronic and paper-based methods were used to gather questionnaires for our study. The electronic questionnaire was primarily shared on social media, where we aimed at collecting a sample of parents and caregivers of children and adolescents without a diagnosis of ASD.

In addition to our online efforts, we collected paper-based surveys in person at two primary locations. These included the John Paul II Paediatric Center in Sosnowiec and the Center for Therapy and Diagnostics of Children with Autism in Biała Podlaska. At these sites, we distributed the surveys to parents of children with ASD diagnoses, which were made by psychiatric specialists based on the DSM-V and ICD-10 criteria, during follow-up visits and handed out questionnaires directly to parents.

Inclusion criteria for our study group included an ASD diagnosis made by psychiatry specialists based on DSM-V and ICD-10 criteria. Exclusion criteria included a history of metabolic disorders and diseases requiring the introduction of diets (e.g., food intolerances). In the control group, the inclusion criteria was a lack of an ASD diagnosis. Exclusion criteria included an ASD diagnosis made by a psychiatry specialist, ASD diagnosis among 1st-degree family members, and a history of metabolic disorders and diseases requiring the introduction of diets (e.g., food intolerances).

By strategically targeting various Facebook groups to form a control group and leveraging in-person data collection at key sites, we were able to assemble a comprehensive and diverse set of data for our study.

The data were collected into Excel and then subjected to statistical analysis in StatSoft Statistica software v. 13 (StatSoftPolska Sp. z o.o., Krakow, Poland). A confidence level = 0.05 was assumed. In order to carry out the statistical analysis, questions were also divided into two groups:

1. Sensory preferences—including questions about the color, structure, temperature, or consistency of food (15 questions).
2. Stereotypical preferences—including questions about the organization of ingredients on the plate (e.g., ingredients mixed/separated on the plate) or type of food (8 questions).

For further analysis, the collected values within the Likert scale were transformed assuming that a value of 3 points describes the “neutral” attitude to a given type of food, and the values obtained by the respondents were converted into a deviation from the value “0”, obtaining the so-called “relative deviation” in the range of preferences (having values −2 to 2) indicating the direction of preference (did the children of the respondents prefer to eat such foods or feel an aversion to them?), and then obtaining their absolute value using the modulus from the obtained number (values 0–2), which made it possible to compare the “strength” of preferences between groups.

The study included 219 people aged 3 to 18 years, of whom 115 had a diagnosis of autism spectrum disorder (52.3%) and 92 had no diagnosis. Twelve children undergoing diagnosis were excluded from the analyses due to the high confounding potential; therefore, 207 people were subjected to the statistical analysis. The age range we established in our study, i.e., setting the lower limit at 3 years, was informed by the common diagnostic practices surrounding autism spectrum disorder (ASD). It is frequently observed that diagnoses are made between the ages of 3 and 18. The early symptoms before the age of 3 years often go unrecognized by parents and specialists. It is important to note that these early symptoms under the age of 3 should be viewed as potential risk indicators for ASD rather than definitive evidence of the disorder. Thus, we set our lower age limit at 3 years to align our study with the age at which these early signs become more readily identified and result in a formal diagnosis.

Due to the aforementioned criteria, 21 individuals without an autism diagnosis and 11 individuals with an autism diagnosis were excluded because their ages did not fall within the specified range. Additionally, 12 individuals who self-reported not having received a complete psychiatric diagnosis yet were also excluded. These individuals demonstrated certain symptoms, yet the absence of a definitive diagnosis risked introducing inconsistencies into our study. Therefore, they were not included in our analysis. In summary, a total of 44 individuals were excluded from the study due to the rigorous application of inclusion and exclusion criteria.

Children with a diagnosis formed the study group, while children who did not have ASD were the control group. In order to better assess the effect of age on food preferences, the analyzed group was also divided into age subgroups according to the most commonly used method of periodization of human development:

1. Middle childhood (preschool age): 4–6 years of age (this group also included people who were 3 years of age;
2. Late childhood (school age): 7–12 years of age;
3. Adolescence: 13–18 years of age.

The age groups of early adolescence, i.e., between 13 and 16 years old, and late adolescence, i.e., from 17 to 18 years old, were merged into one “adolescence” due to the low size of each group. Table 1 presents the distribution of the participants between the created groups.

## 3. Results

**Figure 1:**
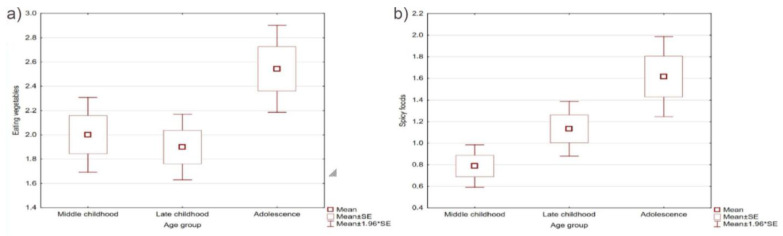
Difference in food preference: (**a**) vegetables and (**b**) spicy foods between created age subgroups.

The mean age of the study group was 8.6 years, while for the control group, it was 9.42 years. The age difference between the groups was not statistically significant, and the age of the respondents alone had no significant effect on the mean relative and absolute deviations of food preference parameters in the Spearman rho analysis. Furthermore, in the analysis broken down by age subgroups using the Kruskal–Wallis ANOVA test, no statistically significant differences were observed in the raw values of the analyzed parameters for all variables, except spicy foods (*p* = 0.02) and the desire to eat vegetables (*p* = 0.02). These differences are presented in Figure 1. Diagnosis and gender did not affect the obtained results.

Females accounted for 37.19% (*n* = 77) of the group. In the study group, this percentage was 25.3% (*n* = 29), and in the control group, it was 52.17% (*n* = 48); the difference was statistically significant according to the chi^2^ test with *p* < 0.05. In contrast to age, the gender of the respondents had a statistically significant effect on the parameters of food preferences in the entire population; however, this result lost its statistical significance when analyzed separately within the study and control groups.

Table 2 shows the descriptive statistics for the raw values of statistically significant variables: mean values, standard deviations, minimum and maximum values, and Mann–Whitney U test results. In the analysis based only on the values translated from the Likert scale, it was shown that there were differences in preferences between children diagnosed with ASD and without a diagnosis in the case of nine variables. It was observed that children with ASD show a clear preference to avoid meals that are sticky or acidic and avoid trying things they do not know (trying novelties). Moreover, after averaging the preference values across all variables, it was shown that, overall, people diagnosed with ASD in all categories scored lower than children without diagnosis subjects: 2.19 vs. 2.45 with *p* = 0.001 in the Mann–Whitney U test.

The average number of “food selectivity traits” in the group of children with and without an autism diagnosis was also analyzed, where answers “does not eat” and “eat reluctantly” to questions about food preferences were treated as a “food selective trait”. In each of the subjects, the abovementioned answers were added together and the average number of traits per child was calculated. The average “food selectivity traits” in those diagnosed with ASD was 9.1 and in those without a diagnosis was 6.9. Autistic people exhibited 24% more of these traits.

The mean relative and absolute deviations from the neutral approach to all analyzed food groups and in terms of sensory and stereotyped preferences in groups of people with ASD and children without a diagnosis were also analyzed. A comparison of deviations in sensory preferences and stereotyped preferences in both groups was also performed with the Wilcoxon test for dependent variables. The results of the analyses are presented in Table 3.

The effect of food color on the willingness to eat a given food was also analyzed, with no significant differences in the absolute degree of preferences between the study and control groups. Interestingly, in both groups, a significant, negative effect of the determined color of food on the child’s preferences was observed (average absolute value > 1 point for all colors) regardless of belonging to the study or control group. In terms of food consistency, the type that showed a difference in absolute deviation was sticky foods, which were significantly less likely to be consumed by people in the study group (−1.09 vs. −0.60, *p* < 0.05).

Subsequently, a logistic regression analysis was carried out to model the predictive effectiveness of the mean relative deviation parameters in the range of preferences for the respondents belonging to the control group. The created model obtained statistical significance with *p* = 0.007, and the obtained parameters were OR = 0.09 for sensory selectivity and OR = 0.56 for stereotypical selectivity. Next, the predictive capacity of absolute values was analyzed, obtaining similar results. Finally, an analysis was carried out for relative and absolute deviation values for average values of variables concerning preferences for the color, taste, and structure of food, obtaining a model devoid of statistical significance. The results of the questionnaire are included in Table 4.

## 4. Discussion

In this study, no statistically significant difference in age was observed between the study and control groups, and the age of the respondents did not significantly affect their food preferences; as a rule, these results coincide with the results of other studies [27,28,29]. Statistically significant differences in the gender distribution of respondents were observed between groups. This was the expected result given the sex ratio in the group of people diagnosed with ASD. However, the observed disproportion did not significantly affect the results obtained due to the lack of relationship between the sex of the subjects and food selectivity shown by other researchers [30].

The analysis of raw parameters representing respondents’ answers to specific questions indicated that, on average, children with ASD show more food selectivity traits compared with children without a diagnosis. Although the severity of food selectivity in neuroatypical children was greater than in children without a diagnosis, it should be emphasized that children without an ASD diagnosis also showed specific food preferences, especially in terms of the specific tastes of the dishes. The obtained results coincide with studies by Tomova et al. [31] and Vernocchi et al. [32], who also described the frequent occurrence of food selectivity in children with autism spectrum disorder but also the presence of food selectivity in some children without diagnosis [32,33].

The obtained data also suggest that children with ASD are more likely to refuse foods with mixed ingredients and dishes in which individual ingredients have come into contact on the plate. The obtained results coincide with the available literature, which also indicates a preference for dishes with a uniform structure and the separation of individual elements of the dish on the plate [30,31]. The effect of a specific appearance of a meal on the willingness to eat it, which was suggested by some authors [29], was not present in the studied group. However, the contact between the products on the plate significantly influenced the food preference in the group of neuroatypical children.

In terms of the taste of the dish, it is worth noting that in the presented study, children with ASD presented a significant aversion to sour food, but for the remaining flavors, there was no statistically significant difference. This is especially interesting in the context of data available in the literature indicating that patients aged 10–19 years with ASD are less accurate in identifying sour and bitter tastes [32]. In terms of specific types of foods, the presented study indicates that children with ASD showed statistically significantly greater aversion to sticky foods compared with children without a diagnosis. Such difficulties may be a manifestation of sensory integration disorders as an axial symptom of ASD. These results may be indirectly consistent with studies showing that children with ASD prefer crispy products [33,34,35,36]. If we assume that stickiness is the inverse of crunchiness, then the research has a common denominator. Neuroatypical people were also less likely to reach for fruit than people without a diagnosis. The study by Cornish et al. [27] indicates the reluctance of children with ASD to eat fruits and vegetables. Research suggests that individuals with ASD may have difficulties with perspective-taking or understanding others’ points of view, which is a cognitive skill often referred to as the theory of mind. This could potentially affect their understanding of the importance of eating fruits and vegetables from their parents’ perspectives [37].

Neuroatypical children presented a significantly greater severity of food restriction than children without a diagnosis, and interestingly, they showed more deviations toward “preferences” than “aversions” to the specific foods. Valenzuela-Zamora et al. [38] describe the phenomenon of a strong aversion to specific foods due to their texture, temperature, taste, color, and smell, with a coexisting preference for specific foods with different characteristics. Among children with ASD, a significantly higher severity of sensory food preferences was observed [5,10]. In addition, people with ASD are less likely to change their habits [39] and may be less likely to give up a “safe” eating regimen including products that were introduced earlier. Food selectivity may be observed in the population of children up to the age of six, regardless of diagnosis; however, in children without a diagnosis, there is a reduction in selectivity after the age of 6, while in neuroatypical children, selectivity may persist throughout life. The expansion of the nutritional repertoire in children without a diagnosis may be explained by the social function of food consumption [40].

Literature reports indicate the occurrence of food selectivity in children with ASD. In a series of studies, the most frequently appearing element that is subject to selectivity is the texture of dishes. Not without significance are also the appearance, taste, smell, and temperature of food. It is also indicated that there is a reluctance to try new dishes and take medication. Available literature data and results obtained in the presented study indicate that the symptom of food selectivity is a heterogeneous phenomenon covering several diagnostic domains. This interpretation of selectivity seems to explain the variability in its prevalence and the nature of the population of children with ASD. The results of the presented study indicate that patients were observed to have both symptoms of sensory aversion to certain food structures and symptoms of stereotypical attachment to certain feeding patterns or organization of dishes on the plate.

Also, the statistical analysis results indicate that although both the sensory and stereotyped domains clearly distinguished themselves in the group of people with ASD, the food parameters associated with stereotypes are significantly more intense than sensory parameters. The logistic regression results also indicate that stereotyped symptoms in terms of selectivity discriminated better against people without diagnosis than neuroatypical people, and their presence was significantly linked to the risk of ASD diagnosis. Perhaps this was due to the fact that stereotypical habits in children with ASD may seem more unusual to the parent, and thus, easier to notice, which may affect the frequency of their reporting.

Although parents of children with ASD are more likely to report food selectivity in their children compared with parents of children without a diagnosis, available literature points out that there is a relationship between an individual’s eating habits and the eating habits of their entire family [41]. In this context, an interesting issue may be the occurrence of a broad autism phenotype (BAP) or simply subclinical features of ASD in the family of the children studied. As these are genetic disorders, it can be assumed that they will be passed from generation to generation within one family [42], determining the dietary choices of its members. In subsequent generations, the eating habits of a given family may be shaped on the basis of the food choices of older family members with subclinical autistic features, which, in turn, may deepen the food selectivity of the child on the spectrum. However, this type of phenomenon requires further research to understand its dietary significance and assess its scale.

Food selectivity leading to excessive consumption of certain types of foods and limiting the consumption of others is not only a problem in terms of finding acceptable meals but can also lead to several health complications. The most visible health risks are gastrointestinal ailments. According to researchers, they may concern up to 88.9% of children and adolescents with ASD, with girls more often affected [29]. In other works, the frequency of gastrointestinal symptoms, such as constipation, diarrhea, abdominal pain, bloating, or gastroesophageal reflux, is estimated to be 23–70% of the neuroatypical population [43].

Difficulties in estimating the real scale of the phenomenon may result from the fact that some of the symptoms may be overlooked, especially in children with ASD with limited verbal communication [44]. Gastrointestinal symptoms in children with ASD are present in all age groups and, as with food selectivity, their degree is not correlated with the severity of ASD symptoms [45]. The most common gastrointestinal symptom in children with ASD is constipation, which can be explained by the aversion to fiber-rich foods, such as vegetables, which was also demonstrated in our study [35]. Furthermore, an additional factor hindering the maintenance of a proper diet may be the co-occurrence of several diseases, e.g., inflammatory bowel disease (IBD), type 1 diabetes, neurological diseases and CNS anomalies, skull structure, and muscular dystrophy, with ASD [46]. The microbiota of children with ASD, just as their eating habits, is different from children without a diagnosis. The core microbiota, i.e., the number and type of bacteria that are common to different individuals [32], is similar in children with and without ASD, but it was found that *Dichelobacter*, *Nitriliruptor*, and *Constrictibacter* may be probable markers of ASD.

There is also evidence indicating that food selectivity in children with ASD might alter gut microbiota, resulting in a more heterogeneous composition compared with their counterparts without food selectivity. Specific bacteria, such as Enterobacteriacaea, Escherichia/Shigella, and Salmonella, were found to be characteristic of “picky eaters”, and might be related to a higher incidence of GI symptoms by acting on the microbiota–gut–brain axis [7].

On the other hand, it was shown that factors such as birth mode, diet, illnesses, and antibiotic treatment in children up to 2 years of age influence the development and type of bacterial colonies in the gut, resulting in a variable composition between individuals. It was demonstrated that the microbiome in children with ASD differs from neurotypical individuals, but there is no consistent evidence regarding the specific changes in the composition of bacterial colonies. The hypothesis concerning pro-inflammatory cytokines produced in gut dysbiosis suggests that they may have a negative impact on brain development, potentially leading to the occurrence of autism spectrum disorder [44].

Research into gut microbiota and its relation to autism spectrum disorder (ASD) suggests potential therapies. Studies show that diet, prebiotics, and probiotics can restore a healthy microbiome. Particularly, probiotics like Lactobacillus species may alleviate gastrointestinal issues and reduce ASD symptoms by correcting dysbiosis [45]. It should be noted, however, that these effects were not due to the effect of the described interventions on the axial symptoms of ASD, but rather by improving the functioning of people with ASD by reducing the number of gastrointestinal symptoms felt.

In the context of ASD, there is evidence to suggest that dysautonomia, or the malfunction of the ANS, may be present. For example, Owens et al. showed in their research notable differences in the autonomic function of individuals with ASD compared with controls. Their findings suggested that ASD is linked to heightened sympathetic activity, as reflected by increased heart rates in both supine and upright positions. The study also reported impaired sympathetic vasoconstriction in ASD, pointing to a complex autonomic dysfunction that intermittently influences cardiovascular and thermoregulatory responses [47]. Dysautonomia can lead to a range of symptoms that may include gastrointestinal (GI) problems [48], which can affect the nutritional status and overall health of the individual.

The complex interplay between ASD, dysautonomia, and digestive function has significant implications for management strategies. For example, if a child with ASD is experiencing GI problems as a result of dysautonomia, dietary modifications alone may not be sufficient to address the issue. In such cases, a more comprehensive approach that includes dietary interventions, medication, behavioral therapies, and possibly interventions to manage the dysautonomia itself may be required.

Studies show that food selectivity can lead to a deficiency in vitamins K, B6, and C, as well as iron, copper, docosahexaenoic acid, and docosapentanoic acid [25], which suggests the potential benefits of supplementation of these ingredients. Additionally, deficiencies in nutrients such as vitamins A, D, and B, as well as calcium and zinc, may be associated with increased fat tissue deposition. Children with ASD more frequently exhibit overweight and obesity based on their BMI. Elevated BMI can result in diseases such as diabetes and cancer. Apart from lifestyle, nutrition, and coexisting medical conditions, the underlying causes for this could be hormonal imbalances and disturbances in gut microbiota. Research indicates that children with ASD have higher levels of anorexigenic leptin compared with peers with a similar BMI. It was demonstrated that ASD is more often diagnosed in children who experience rapid weight gain and have elevated leptin levels. Children with ASD have more problems with nighttime sleep and increased daytime sleepiness, which can result in reduced physical activity during the day and weight gain [44]. In another review study, it was observed that girls with autism spectrum disorder (ASD) experience more feeding problems compared with girls with a similar BMI and age without ASD, where an issue was identified in 11% of the 52 girls with ASD, which included dieting, self-starvation, and purging. Patients with ASD showed a higher interest in topics related to food and oral control. Additionally, mothers of girls with ASD also reported their daughters being interested in these topics. Among children with ASD, pica (eating non-food items) is more frequently observed than in children without ASD [4].

In addition, the increasingly discussed impact of the microbiome on ASD also encourages parents to try various dietary interventions. Some studies indicate that up to 25% of people with ASD use dietary interventions, but this percentage may vary depending on the country in which the study was conducted. The results regarding the effectiveness of dietary interventions, such as restrictive diets (most often gluten-free and casein-free), and dietary supplements, such as vitamins, minerals, amino acids, omega-3s and herbal compounds, in ASD are still controversial [49]. This meta-analysis showed that in people with ASD, dietary supplementation with omega-3 acids and vitamin supplementation was more effective than a placebo in improving specific symptoms, e.g., omega-3 supplementation worked better than a placebo at improving language function and social functioning, while vitamin supplementation reduced stereotypes and had a positive effect on behavioral symptoms. The cited meta-analysis also drew attention to the low size of the effect of the analyzed studies compared with a placebo. Thus, they suggested that dietary supplements may exert a nonspecific and minor effect on ASD [50].

## 5. Conclusions

The current research presents several potential limitations that must be taken into account when interpreting the results. First, there may have been a sampling bias, as participants were not selected at random. Instead, they were drawn from specific institutions, which may limit the applicability of the findings to broader, more diverse populations. Therefore, our sample might not be representative of all parents of children with or without ASD. Second, the study relied on self-reported data from parents or guardians. As such data are subjective, they could be influenced by recall bias, misinterpretation of questions, or social desirability bias. There may also be disparities in parents’ understanding and interpretation of their child’s food preferences. Third, the research did not account for the variability in ASD severity between participants. The heterogeneity of ASD could potentially impact the findings and should be considered in future research.

The results of the presented study indicate a higher incidence of food selectivity and its greater severity in children with ASD than in children without a diagnosis. It should be pointed out that while food selectivity is common in children with ASD, neurotypical children also present food selectivity to some extent, especially in the context of taste aversions, which were more unambiguous in the group of people without a diagnosis. In addition, food preferences in children with ASD can be varied. Dietary habits of the entire family may also influence food selectivity or lead to other nutritional deficiencies or intolerances. The results obtained in the present study indicate that the symptoms of food selectivity constitute a heterogeneous phenomenon covering several diagnostic domains. This interpretation seems to explain well the variability in prevalence and the nature of selectivity in the population of children with ASD. Although it is partially linked to the sensory domain, food selectivity in the neuroatypical population seems to be predominantly stereotypical in its nature.

## Figures and Tables

**Table 1:** Distribution of the participants between the age groups created; ^1^ percentage of the entire subgroup.

Group	*n*	ASD Group (Females)	Control Group (Females)
Middle childhood	69	42 (7) 60% ^1^	27 (14) 39%
Late childhood	91	51 (12) 56.4%	40 (25) 43.6%
Adolescence	47	22 (10) 46.8%	25 (9) 53.2%

**Table 2:** The preference for specific characteristics of food in children with and without ASD.

Variable	Average ASD Group	95%CI ASD Group	Mean Control Group	95%CI Control Group	*p*-Value
Sticky foods	1.71	[1.5–1.91]	2.31	[2.1–2.51]	0.0000
Foods with a certain appearance	2.43	[2.23–2.64]	2.78	[2.58–2.97]	0.025
Foods with a specific flavor	2.05	[1.86–2.25]	3.09	[2.89–3.28]	0.0017
Sweet foods	3.16	[2.95–3.37]	3.52	[3.37–3.68]	0.026
Acidic foods	1.56	[1.32–1.8]	2.09	[1.83–2.35]	0.0002
Foods with mixed ingredients	1.78	[1.59–1.98]	2.43	[2.22–2.63]	0.0000
Vegetables	1.83	[1.58–2.08]	2.38	[2.13–2.64]	0.0002
Fruit	2.35	[2.1–2.61]	2.96	[2.73–3.18]	0.001
Ingredients in contact with each other on the plate	1.82	[1.62–2.01]	2.34	[2.15–2.52]	0.001
Trying new things	1.36	[1.16–1.56]	2.36	[2.15–2.58]	0.0000

**Table 3:** Mean deviations from neutral (=0) for all analyzed foods and sensory and stereotyped preferences; MED—median. * *p*-value for comparison of mean absolute sensory preference vs. stereotypical preference.

Variable	ASD Group Av (95%CI)	ASD Group MED	Control Group Av (95%CI)	Control Group MED
Mean relative deviation	0.10 (0.01–0.19)	0.08	−0.08 (−0.15–(−0.02))	−0.08
Mean relative deviation—sensory preferences	−1.08 (−1.17–(−0.98))	−1.12	−1.21 (−1.29)–(−1.1))	−1.25
Mean relative deviation—stereotypical preferences	−0.85 (−0.95–(−0.74))	−0.91	−1.07 (−1.15–0.99)	−1.08
Mean relative deviation—taste	−1.07 (−1.24–(−0.95))	−1	−1.26 (−1.39–(−1.12))	−1
Mean absolute deviation	1.10 (1.01–1.19)	1.08	0.91 (0.84–0.97)	0.91
Mean absolute deviation—sensory preferences	0.91 (0.82–1.01)	0.87	0.78 (0.70–0.86)	0.75
Mean absolute deviation—stereotypical preferences	1.14 (1.04–1.25)	1.08	0.92 (0.84–1)	0.91
Mean absolute deviation—taste	1.41 (1.3–1.51)	1.5	1.27 (1.17–1.37)	1.33
*p*-value (Wilcoxon) *	<0.05		<0.05	

**Table 4:** Questions from the questionnaire with the results that produced *p* > 0.1.

Variable	Mean Control Group	95%− Control Group	95%+ Control Group	Mean ASD Group	95%− ASD Group	95%+ ASD Group
Hard foods	2.534091	2.309332	2.758849	2.169643	1.947829	2.391457
Sticky foods	2.307692	2.102481	2.512904	1.707965	1.504572	1.911357
Foods that are easy to chew	2.978022	2.802462	3.153582	2.780702	2.598029	2.963375
Crumbly foods	2.923913	2.739106	3.10872	2.710526	2.516584	2.904469
Crispy foods	3.010989	2.820888	3.20109	2.947826	2.763351	3.132301
Foods of certain appearance	2.777778	2.582261	2.973294	2.432432	2.228541	2.636324
Foods of certain taste	3.087912	2.894909	3.280916	2.598214	2.387243	2.809186
Sweet foods	3.522222	3.367679	3.676765	3.157895	2.947588	3.368201
Salty foods	2.728261	2.48054	2.975982	2.54386	2.344622	2.743098
Sour foods	2.087912	1.830686	2.345138	1.558559	1.317026	1.800091
Bitter foods	1.144444	0.910411	1.378478	0.920354	0.737081	1.103627
Spicy foods	1.208791	0.971305	1.446278	1.070796	0.84981	1.291783
Foods of certain smell	2.296703	2.07829	2.515116	2.054545	1.858198	2.250893
Foods of certain temperature	2.32967	2.12834	2.531	2.267857	2.088399	2.447315
Warm foods	2.67033	2.491838	2.848821	2.415929	2.240289	2.591569
Foods of room temperature	2.717391	2.551131	2.883652	2.649123	2.494995	2.803251
Cold foods	2.25	2.044907	2.455093	2.106195	1.911082	2.301307
Foods of certain color	2.461538	2.264435	2.658642	2.368421	2.183256	2.553586
Red/orange foods	2.406593	2.200712	2.612475	2.217391	2.017438	2.417344
Green foods	2.163043	1.963418	2.362669	1.965217	1.760606	2.169829
White foods	2.274725	2.110254	2.439196	2.156522	1.987726	2.325317
Yellow foods	2.362637	2.199784	2.525491	2.192982	2.010112	2.375853
Grey foods	1.879121	1.673574	2.084668	1.884956	1.698977	2.070935
Moist foods	2.222222	2.039745	2.404699	2.214286	2.0323	2.396271
Dry foods	2.450549	2.261035	2.640064	2.605263	2.414657	2.79587
Foods of certain texture	2.456522	2.298735	2.614309	2.330357	2.148441	2.512273
Liquid foods	2.593407	2.420613	2.7662	2.5	2.31651	2.68349
Semi-liquid foods	2.358696	2.18345	2.533942	2.315789	2.119361	2.512217
Solid foods	2.641304	2.474321	2.808287	2.622807	2.447572	2.798042
Foods with mixed ingredients	2.428571	2.222304	2.634839	1.780702	1.58514	1.976264
Foods in which ingredients are separated	2.48913	2.314119	2.664142	2.392857	2.209267	2.576447
Eating vegetables	2.384615	2.130934	2.638296	1.831858	1.584152	2.079565
Eating fruits	2.956522	2.733194	3.17985	2.350877	2.095546	2.606208
Foods pressed through a sieve	2.315217	2.126136	2.504299	2.026087	1.798574	2.2536
Foods in which ingredients have come into contact	2.336957	2.152039	2.521874	1.817391	1.620759	2.014023
Trying foods for the first time	2.362637	2.146393	2.578882	1.356522	1.155958	1.557086
Vitamins and supplements	2.573034	2.3675	2.778567	2.052632	1.81096	2.294303

## Data Availability

The data presented in this study are available on request from the corresponding author. The data are not publicly available due to privacy reasons.

## Supplementary Materials

The following supporting information can be downloaded at: https://www.mdpi.com/article/10.3390/jcm12175469/s1, Supplementary Table S1: author’s questionnaire.
