# Supplementary material for: Patterns of Food Selectivity among Children with Autism Spectrum Disorder
Source: J Clin Med. 2023 Aug 23;12(17):5469. doi: 10.3390/jcm12175469 (PMC10488249; doi:10.3390/jcm12175469)
Supplement: Supplementary file 1 [file jcm-12-05469-s001.zip › jcm-2521654-SI.pdf]

Table S1: Author’s questionnaire.

Ladies and Gentlemen,
we are students of Medical University of Silesia in Katowice and as members of Student’s scientific association at the Department of Psychiatry and Psychotherapy of Developmental Age we are preparing a paper on patterns of food selectivity among children with autism spectrum disorder. Following questionnaire is anonymous and voluntary. Collected data is going to be used exclusively for research purposes. Thank you for completing this questionnaire.

-Please select the answer:
-Child’s gender: Male/Female/ Other/I would rather not say.
-Has your child been diagnosed with autism: yes/ no/ during diagnosis.
-Child’s age (in ages) … .
-Size of the dwelling place: countryside/ a town of population up to 50 thousand residents / a town of population between 50 and 100 thousand residents / a city of population between 100 and 250 thousand residents / a city of population above 250 thousand residents.
- Please select the answer which best describes the child’s willingness to eat regarding following traits:

	does not eat	eats reluctantly	does not affect the willingness to eat	eats willingly	eats very willingly
hard foods					
sticky foods					
foods that are easy to chew					
crumbly foods					
crispy foods					
foods of certain appearance					
foods of certain taste					
sweet foods					
salty foods					
sour foods					
bitter foods					
spicy foods					
foods of certain smell					
foods of					

certain temperature					
warm foods					
foods of room temperature					
cold foods					
foods of certain color					
red/orange foods					
green foods					
white foods					
yellow foods					
grey foods					
moist foods					
dry foods					
foods of certain texture					
liquid foods					
semi-liquid foods					
solid foods					
foods with mixed ingredients					
foods in which ingredients are separated					
eating vegetables					
eating fruit					
foods pressed through a					

sieve					
foods in which ingredients have come into contact					
trying foods for the first time					
vitamins and supplements					

Please describe any other atypical behaviors related to food that you have observed in your child:
………………………………………………………………………………………………………..……
……………………………………………………………………………………………………….….
……………………………………………………………………………………………………….….
